# Text recycling in health sciences research literature: a rhetorical perspective

**DOI:** 10.1186/s41073-017-0025-z

**Published:** 2017-02-16

**Authors:** Cary Moskovitz

**Affiliations:** Thompson Writing Program, Duke University, Durham, NC USA

**Keywords:** Text recycling, Plagiarism, Self-plagiarism, Paraphrasing, Publication ethics

## Abstract

The past few years have seen a steady rise in the number of health science journals using plagiarism detection software to screen submitted manuscripts. While there is widespread agreement about the need to guard against plagiarism and duplicate publication, the use of such tools has sparked debate about text recycling—the reuse of material from one’s prior publications in a new manuscript. Many who have published on the topic consider all uses of text recycling anathema. Others argue that some uses of recycling are unavoidable and sometimes even beneficial for readers. Unfortunately, much of this discourse now merely repeats dogmatic assertions. I argue that progress can be made by acknowledging three points: First, citation standards for research writing in the health sciences will not mirror those of the humanities. Second, while it is impossible to draw a definitive line between appropriate and inappropriate uses of text recycling, some uses of the practice lie clearly on the legitimate side. Third, the needs of editors for information regarding recycled text are different from those of readers. Ultimately, calls for rewording and citation as alternatives or fixes for text recycling are unlikely to prove satisfactory to either readers or editors.

A response to this article can be found using the following link: http://researchintegrityjournal.biomedcentral.com/articles/10.1186/s41073-017-0026-y.

## Background

In recent years, increasing numbers of health science journals have begun testing submitted manuscripts with plagiarism detection software such as Ithenticate. While there is widespread agreement about the need to guard against plagiarism and duplicate publication, the use of such tools has also fueled debate about “text recycling”—the reuse of material from one’s prior publications in a new work. In the past decade alone, editorials, letters, and essays addressing text recycling have been published in health science journals literally across the globe—including such countries as Nepal [1], Spain [2], the UK [3], Bosnia [4], Iran [5], Croatia [6], India [7], Canada [8], and the USA [9]. The debate has also engaged a remarkably broad range of health care specialties. Journals that have published editorials or other position pieces on text recycling or “self-plagiarism” include (among others) *Indian Journal of Sexually Transmitted Diseases* [7], *Journal of The American Academy of Dermatology* [9], *Journal of General Internal Medicine* [10], *International Urogynecology Journal* [11], *Skeletal Radiology* [12], *Anesthesia* [13], and the *Journal of Medical Toxicology* [14].

Many of these pieces argue that text recycling is not appropriate under any circumstances. Those who hold the contrary view often argue that rewording prose merely to avoid charges of self-plagiarism is unreasonable. This debate seems to have reached a stalemate. Having studied text recycling and this debate from a writing studies perspective, I see two considerations that might help to advance the conversation: understanding that conventions for source attribution and text reuse are contextual rather than universal, and recognizing that readers and editors have different needs for information regarding sources and text reuse.

## The assumption of universal norms

Confusion among editors and authors regarding text recycling has led some professional organizations to take up the matter, and at least three high-profile organizations have established that *some* uses of text recycling are indeed legitimate. According to the most recent edition of the Publication Manual of the American Psychological Association [15], there are “circumstances (e.g., describing the details of an instrument or an analytic approach) under which authors may wish to duplicate without attribution (citation) their previously used words, feeling that extensive self-referencing is undesirable or awkward. When the duplicated words are limited in scope, this approach is permissible.” Similarly, BioMed Central collaborated with the Committee on Publication Ethics (COPE) to address the matter in 2013 [16], resulting in a set of guidelines for editors, “How to deal with text recycling” [17]. These guidelines not only state that text recycling is sometimes acceptable but that in some cases it may be *preferred*:

Some degree of text recycling in the background/introduction section of an article may be unavoidable, particularly if an article is one of several on a related topic. Duplication of background ideas may be considered less significant *or even considered desirable* … Use of similar or identical phrases in methods sections where there are limited ways to describe a method is not unusual; in fact text recycling may be unavoidable when using a technique that the author has described before and it *may actually be of value* when a technique that is common to a number of papers is described… Some degree of text recycling may be acceptable in the discussion; ([1, 2, 17], emphasis added)

Nevertheless, numerous editorials, letters, blog posts, and so on reinforce the idea that the practice is *intrinsically* problematic and unethical. Some, like an editorial from the *Canadian Journal of Hospital Pharmacy*, state their position in a straightforward manner: “Repeating the same passages verbatim in multiple papers should not be considered acceptable” [8]. Others, however, infer intellectual or ethical shortcomings in any author who recycles text. An editorial published in the *Lancet* in 2011 states that “experienced authors with a large publication list from reputable institutions are expected to know that recycling past material is inappropriate” [3]. Another editorial, published this year in the *Journal of the American Association of Nurse Practitioners*, declares that “editors are not unreasonable in their demands for ‘original’ material,” and that “We need to reserve recycling to our trash disposal practices and not our literature” [18]. And for one last example, a response from the Managing Editor of the *American Journal of Preventive Medicine* to the then-recently-posted COPE forum on text recycling:

I’ve been “iThenticating” all revised papers for several years now, and am continually frustrated by self-plagiarism. I now have two lines that I repeat to our editors on a regular basis: “Self-plagiarism is, by its very name, plagiarism”; and “you’d think that researcher/authors with MDs and PhDs would be bright enough to know how to reword [19].”

Those who are fundamentally opposed to text recycling often argue against the practice on one of two grounds: that it thwarts “standard” citation practices or that it violates a contract between writer and reader. Both arguments rest on the assumption that universally applicable norms for source attribution and text reuse exist. A particularly important example of such arguments is Miguel Roig’s widely referenced essay “Avoiding plagiarism, self-plagiarism, and other questionable writing practices: A guide to ethical writing” [20], which is widely cited and referenced by health scientists, journal editors, and educational institutions as an authoritative source on text recycling and self-plagiarism. A Google Scholar search shows over 100 such references, and the Office of Research Integrity (ORI) at the U.S. Office of Health and Human Services even houses this essay as a resource on its website. ORI replaced the 2006 version of this essay on its website with a revised version in 2016 [21]; however, further references to this document in the present essay refer to the 2006 version [20] as this version has informed current opinions regarding text recycling and self-plagiarism. The widely cited 2006 version of this essay urges authors to “adhere to the spirit of ethical writing and avoid reusing their own previously published text, unless it is done in a manner consistent with *standard scholarly conventions* (e.g., by using of quotations and proper paraphrasing)” [emphasis added]. Similarly, an opinion piece published in the *Archives of Iranian Medicine* insists “we have to comply with the universally-accepted definitions of plagiarism” [5].

Yet contrary to such statements, there are no such universal standards. The fallacy stems from believing that conventions for source attribution in the humanities apply equally to all genres and contexts. Such confusion is understandable: the writing instruction most of us receive—in elementary school, secondary school, and even college English courses—is provided by teachers trained in humanities disciplines, and they tend to teach their fields’ citation practices as if they *were* generic [22, 23]. But when we look beyond school writing and humanities scholarship, the norms vary considerably—which should be evident to anyone familiar with writing in law, journalism, or business. And we see it within the classroom context as well: plagiarism policies of universities are often repeated verbatim in course syllabi without the use of quotation marks and often without attribution.

Similarly erroneous is the argument that text recycling is inherently misleading to readers. The “Avoiding Plagiarism” essay [20] includes what is certainly the most widely disseminated argument of this kind, asserting that recycling violates an “implicit contract” between reader and writer:

[E]thical writing…entails an implicit contract between reader and writer whereby the reader assumes, unless otherwise noted, that the material was written by the author, is new, is original… Because [Methods] sections are often highly technical and can be laborious to write, authors of multiple papers using the same methodology will sometimes recycle text with little or no modification from a previously published paper and use it in a new paper. Technically, [adhering to] the ‘implicit contract’ between reader and writer embodied in the concept of ethical writing and to the strict rules of proper scholarly conduct, s/he would need to put any verbatim text from the method section in quotation marks and appropriately paraphrase any other recycled text that is not placed in quotations.

Argument by reference to such an implicit contract has become dogma of sorts, showing up in many arguments against all uses of text recycling (see for example, [5, 24, 25]). (Roig himself continues to use the contract argument against recycling in his more recent work [26].)

The concept of an implicit contract between writers and readers does indeed exist in discourse on writing. However, the way Roig and others use the concept—as a precept against text recycling—appears to be an unwarranted extrapolation. The term seems to originate with Abrams reference book, *A glossary of literary terms* [27]. Abrams idea of such a figurative contract was in reference to reader expectations of literary genres; for example, such a contract would imply that the reader of a work of historical fiction should be safe to assume that the author had not invented or altered major relevant historical events. The reader-writer contract is also discussed at length in Tierney and LaZansky’s essay “The Rights and Responsibilities of Readers and Writers: A Contractual Agreement” [28] which summarizes the writer’s obligations this way: “[A]uthors have a responsibility to their audience—a responsibility which necessitates that written communications be relevant, sincere, and worthwhile.” One might reasonably say that according to such a contract, a newly published scientific report should present new science. But neither the Abrams nor the Tierney/LaZansky essay states or infers any universal expectation regarding originality of prose. Quite to the contrary, both emphasize the need for writers to consider the particular context of their readers.

A foundational concept in the fields of contemporary rhetoric and writing studies is that the conventions for all types of writing are established by (and frequently changed by) members of that community’s writers and readers. In direct contradiction to the idea of universal norms for source attribution, genre studies scholarship has established that such norms are “local”—determined by each discourse community [28, 29]. As for the health sciences, conventions for recycling text are clearly distinct from those of the humanities. In addition to the guidelines published by BioMed Central and the American Psychological Association quoted above, an informal poll taken by editors at the *Journal of General Internal Medicine* showed that “many experts . . . are fine with around 10% recycling of verbiage, some even arguing for the benefits of repeating complex methods verbatim” [10]. Even Roig acknowledged that the standards he discussed in his essay are not normative for the sciences. Following his description of “proper scholarly conduct” as quoted above, he notes: “Curiously, such practice is seldom, if ever, followed in these instances. Instead, what seems to have become a routine practice for authors is to recycle, with some minor modifications, substantial portions of these sections” [20].

At this point, those opposed to text recycling might ask why authors should not use the humanities conventions for paraphrasing (rewording), quotation, and citation anyway. After all, can not everyone just avoid potential concerns about recycling by either paraphrasing or formally quoting and citing verbatim passages?

## Rewording

Let us take the matter of rewording first by considering a case study: a sequence of articles reporting on trials of the RTS, S/AS01 malaria vaccine [30–32]. I choose this case because the health care research community would clearly *expect* the researchers to publish their findings at multiple stages during the research—from early safety studies to short-term, small-scale trials, and to longer, larger trials if the early studies are promising. (They were.) Since each of these studies is clearly publication-worthy based on scientific merit, we can set aside concerns about “salami-slicing” or duplicate publication and focus on the writing itself.

**Fig. 1:**
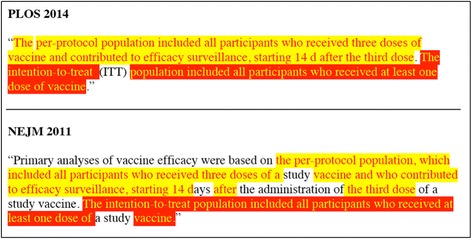
Passages from the Methods sections of two papers in the malaria case study: New England Journal of Medicine [30] and PLOS Medicine [32]

Deciding whether to paraphrase or recycle text in these situations is clearly a complex, nuanced matter. Unlike the author of literary works or other essayistic prose, a reasonable argument can be made for keeping some wording the same across a sequence of related scientific articles. The question at hand then is not “Should recycled text *ever* be replaced with paraphrased text?” (Often that is the appropriate decision.) The question is “Is it *always* preferable to reword rather than recycle?” In answer to this question, consider Fig. 1, which contains excerpts from two papers in the malaria case study. Given the nature of this particular material, I believe that readers are better served by retaining the wording as much as possible.

We should also consider the rewording option not only in principle but also in practice. Many editorials encourage the rewording of all recycled text—but I wonder whether the authors of these editorials have carefully considered the likely result. Here is another passage from the 2011 *New England Journal of Medicine* paper [30]:

During 12 months of follow-up in the first 6000 children in the older age category, the incidence of the first or only episode of clinical malaria meeting the primary case definition was 0.44 per person-year in the RTS, S/AS01 group and 0.83 per person-year in the control group, resulting in a vaccine efficacy of 55.8% (97.5% confidence interval [CI], 50.6 to 60.4)

**Fig. 2:**
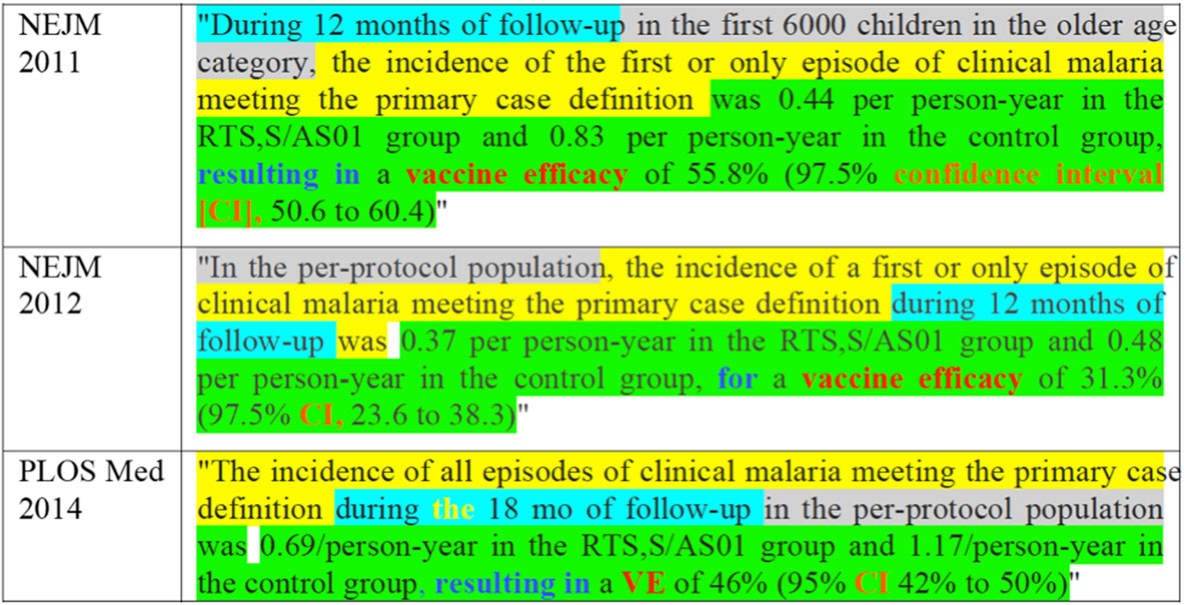
Three versions of a passage defining clinical malaria cases from a sequence of reports published in the New England Journal of Medicine [30, 31] and PLOS Medicine [32]

As shown in Fig. 2, the authors *did* reword this passage in subsequent articles [31, 32].

Note the rearrangement of passages, substitution of synonyms, and so on. We see similar substitutions and rearrangements throughout this chain of articles: “37.5 °C or higher” becomes “a temperature ≥37.5 °C”; parasite density is presented in units of “per cubic millimeter,” “/mm^3^” and “/μL”; and so on. I do not know whether this is the kind of paraphrasing these editorials hope for, but when scientists are pressed to rework accurate and perfectly effective prose merely to avoid being flagged by plagiarism detection software, it is what we should expect: superficial and arbitrary changes that ultimately make reading harder for those following the line of research. To be clear, I am not suggesting that scientists should never paraphrase their own writing; paraphrasing often is the best choice. But from a communication perspective, rewording informational statements makes sense only when the context of the writing changes in some way—different audiences, different genres, or different purposes. The type of situation I am considering here—papers from studies that build on one another—typically involve the *same* type of writing intended for the *same* audience.

As for the ethics, arguments for preferring rewording to text recycling are often made on the basis that recycling is deceptive while rewording is forthright. But consider again Figs. 1 and 2: Given the clear need for the same information in multiple papers, there is no basis for assuming that authors who would choose to recycle text in such situations would do so with intent to mislead. And is rewording one’s previously published prose really more ethical? After all, the reworded version is not really *new* text but recycled text in disguise—an illusion of difference where no meaningful difference exists.

One last point related to rewording: We all want editors to be able to screen submission for real, intentional plagiarism and duplicate or redundant submissions. Encouraging authors to reword rather than recycle text might make it harder for editors to detect the cases that really do compromise scientific integrity.

## Citation and quotation

Those who have offered guidance on how authors should handle text recycling in their work almost always state that the sources of recycled material should be cited (see for example refs [15, 17, 33]). Many directly state or imply that a parenthetical citation to that source of the recycled material supplemented, perhaps by a clause referencing the source, (e.g., “As noted in a previous article…”), would suffice to resolve any concerns about recycling.

Yet there are a number of problems with this assumption. First, as is the case for the malaria vaccine studies, authors usually cite the sources of recycled material anyway—in their discussion of relevant prior research. How can authors establish that an article cited for its content is also being cited as the source of recycled material? Similarly, if authors follow citation conventions from the humanities, these sources should be cited regardless of whether the authors have paraphrased or recycled passages. How are readers or editors to know from the reference whether passages have been recycled or paraphrased? Neither do citations identify which specific parts of the paper were recycled. (And there is also the matter of increasing self-citations: Would the scientific community condone large numbers of additional references to one’s own publications for the purpose of acknowledging recycled text?)

So how about treating recycled passages as quotations? This practice is often suggested, as in the *Lancet* editorial: “[W]hether [text recycling] is misconduct depends on the extent of the duplicate text and authors’ circumstances. Inexperienced authors whose first language is not English might just need education about the use of quotation marks and citations” [3]. And, as quoted above, Roig [20] makes an even more direct assertion about the use of quotations as appropriate for this purpose. Yet serious consideration of using quotation marks to identify recycled material in research reports shows that it is not a viable option. Most importantly, the very nature of quotation marks (and block indentation) is to draw readers’ attention to the quoted words. For recycled text, this would be rather odd if not downright bewildering. Also, articles that contain recycled text rarely have only a single, contiguous block of recycled material. Instead, we often find bits of recycled material scattered throughout Introduction and Methods sections. Following this advice would mean that readers would encounter dozens of pairs of quotation marks or blocks of indented text scattered throughout the paper. I cannot imagine that editors or readers would find this acceptable in a scientific article.

## Meeting the needs of editors

It is inevitable that health science researchers will regularly confront situations in which they need to include essentially the same material in a series of related publications. For the most common situations—presenting background material and describing methods—organizations such as the American Psychological Association, BioMed Central, and COPE have established that some amount of text recycling is indeed appropriate. The challenge is then to determine an appropriate protocol. As I have explained, humanities norms for attribution do not offer suitable approaches because they do not allow authors to effectively identify recycled text without also altering the manuscript in undesirable ways: Quotation marks draw inappropriate attention to the recycled text and would generally be considered inappropriate for scientific reports; citations do not distinguish between paraphrased and recycled text nor between sources of recycled material and references that are included for their content.

We have reached the point, it seems, where new approaches specifically designed to address text recycling are warranted. Until now, those who have addressed the challenges posed by text recycling have assumed that the needs of those involved in the editorial process and the needs of those who are only “consumers” of the research papers must be met in a single, identical manuscript. As a first step, we might recognize that, as gatekeepers for their journals, editors have different responsibilities and therefore different needs than readers. What is needed is a mechanism by which authors *can show editors* which parts of their manuscripts have been recycled, without compromising the text itself.

Highlighting—the method I have used for this purpose with my students for nearly a decade—is worth considering. If authors highlight recycled passages in manuscripts, citations and explanations for highlighted (recycled) material could then be documented directly in a separate memo to the editor. Highlighting recycled text eliminates concerns about duplicity—for it announces the presence of recycled material in a detailed and fully transparent way. Unlike quotation marks, highlighting recycled text does not interfere with the manuscript’s syntax and is easily removed during the editorial process. As for readers, they rely on the editorial process to ensure that published papers are scientifically sound and relevant. Surely readers can trust them to ensure that any recycled material is within acceptable limits.

## Conclusion

The health care research community is clearly in need of better text recycling guidelines—for both editors and authors. While the BioMed Central/COPE guidelines are a productive step forward, editors and authors need something more direct and specific. Getting there will require shared understanding on three points: First, citation standards for health care research writing will not mirror those of the humanities. Second, while it is impossible to draw a definitive line between appropriate and inappropriate uses of text recycling, *some* uses of the practice lie clearly on the legitimate side. Third, editors need more specific information about the presence of recycled text, but this information need not be included the published manuscript.

These understandings would allow journals to set as policy that *some kinds and amounts* of recycling could be included. Although no single, universal threshold is feasible, a reasonable starting point for deliberation might be, say, 10% of Introduction and Methods sections and 5% elsewhere. If authors should feel the need to exceed such thresholds in specific instances, they could work it out through open and direct communication with the editor.

Setting an established threshold would make things considerably easier for both editors and authors. Given the cultural norms of writing in the health sciences, the inclusion of recycled material in manuscripts is inevitable for the foreseeable future. With such thresholds, editors would no longer have to make difficult, individualized judgments for each bit of recycled material; instead, they could focus their attention on cases involving large amounts of recycled material—the papers that are most likely to be worth investigating. As for the many authors who have legitimate needs for repeating a limited amount of material from prior papers: no longer needing to worry about being snared by Ithenticate and its kin, authors could cease the inane practice of replacing words with synonyms and rearranging clauses in a manner resembling the process of a middle-school student writing a “research paper” from a Wikipedia article.

Some readers of this essay will object to the setting of specific, quantitative thresholds, on the grounds that the quantity of recycled material is not a good measure of its appropriateness. This concern is legitimate. The acceptability of any particular bit of recycled text does indeed depend on its rhetorical function; technical information about a statistical method is judged differently than a statement of findings or implications. Yet if the manuscript in question has been determined by the editor to be worthy of publication on its scientific merits, it is hard to imagine the objection of readers regarding small amounts of recycled text of specific kinds, especially rehearsing relevant literature or providing technical information. And if the recycled text is highlighted as described above, editors can decide if specific instances even below these thresholds are problematic and address them with the authors.

Health care researchers have repeatedly seen what happens when new diagnostic tools get widely implemented without due consideration of how findings should be interpreted. As the use of Ithenticate and other digital plagiarism detection systems rapidly becomes standard practice in healthcare research publishing, care should be taken to ensure that this new tool is used thoughtfully and cautiously. Moving forward without a specific, sensible protocol will cause editors and researchers wasted effort and avoidable frustration.

## References

[1] Adhikari N (2010). Avoiding plagiarism and self-plagiarism. J Nepal Paediatr Soc.

[2] Agud JL (2014). Fraud and plagiarism in school and career. Rev Clin Esp (English Edition).

[3] Kleinert S (2011). Checking for plagiarism, duplicate publication, and text recycling. Lancet.

[4] Mehic B (2013). Plagiarism and self-plagiarism. Bosn J Basic Med Sci.

[5] Farrokhi F (2009). Plagiarism: where unawareness makes a lame excuse. Arch Iran Med.

[6] Habibzadeh F, Shashok K (2011). Plagiarism in scientific writing: words or ideas?. Croat Med J.

[7] Carver JD, Dellva B, Emmanuel PJ, Parchure R (2011). Ethical considerations in scientific writing. Indian J Sex Transm Dis.

[8] Tisdale JE (2009). Integrity in authorship and publication. Can J Hosp Pharm.

[9] Dellavalle RP, Banks MA, Ellis JI (2007). Frequently asked questions regarding self-plagiarism: how to avoid recycling fraud. J Am Acad Dermatol.

[10] Kravitz RL, Feldman MD (2011). From the editors’ desk: self-plagiarism and other editorial crimes and misdemeanors. J Gen Intern Med.

[11] Lose G (2011). Plagiarism. Int Urogynecol J.

[12] Berlin L (2009). Plagiarism, salami slicing, and Lobachevsky. Skelet Radiol.

[13] White SM (2011). Self-plagiarism. Anaesthesia.

[14] Bird SB, Sivilotti ML (2008). Self-plagiarism, recycling fraud, and the intent to mislead. J Med Toxicol.

[15] American Psychological Association (2010). Publication manual of the American Psychological Association.

[16] Committee on Publication Ethics (COPE). Text Recycling Guidelines [Internet]. Available at: http://publicationethics.org/text-recycling-guidelines. Accessed 9 Jun 2015.

[17] BioMed Central: How to deal with text recycling. Available at: http://media.biomedcentral.com/content/editorial/BMC-text-recycling-editorial_guidelines.pdf. Accessed 9 Jun 2015.

[18] Pierson C (2016). Recycling is good for trash but unacceptable for scientific literature. J Am Assoc Nurse Pract.

[19] Seidman, C. Comment, posted 22/2/2013 as response to COPE Text Recycling Guidelines [Internet]. Available at: http://publicationethics.org/text-recycling-guidelines. Accessed 9 Jun 2015.

[20] Roig M. Avoiding plagiarism, self-plagiarism, and other questionable writing practices: A guide to ethical writing | ORI - The Office of Research Integrity (2006 version). Available at: https://ori.hhs.gov/images/ddblock/plagiarism.pdf. Accessed 7 Dec 2016.

[21] Roig M. Avoiding plagiarism, self-plagiarism, and other questionable writing practices: a guide to ethical writing (2015 version). Available at: https://ori.hhs.gov/sites/default/files/plagiarism.pdf. Accessed 7 Dec 2016.

[22] Dowdey D. Citation and Documentation Across the Curriculum. ResearchGate [Internet]. 1992. Available at: https://www.researchgate.net/publication/251845333_Citation_and_Documentation_Across_the_Curriculum. Accessed 16 Jun 2016.

[23] Wolfe J, Olson B, Wilder L (2014). Knowing what We know about writing in the disciplines: a new approach to teaching for transfer in FYC. WAC Journal.

[24] Viale PH (2012). Avoiding plagiarism in professional writing. J Adv Pract Oncol.

[25] Shah A (2012). Plagiarism: the bête noire of scientific communication. Indian J Chest Dis Allied Sci.

[26] Roig M (2014). Critical issues in the teaching of responsible writing. J Microbiol Biol Educ.

[27] Abrams MH. *A Glossary of Literary Terms, Seventh edition*. Fort Worth: Harcourt Brace College Publishers. 1999. [cited 2016 Jun 22]. Available from: http://www.ohio.edu/people/hartleyg/ref/abrams_mh.pdf

[28] Tierney RJ, LaZansky J (1980). The rights and responsibilities of readers and writers: a contractual agreement. Lang Arts.

[29] Hyland K (1999). Academic attribution: citation and the construction of disciplinary knowledge. Appl Linguist.

[30] The RTS SCTP (2011). First results of phase 3 trial of RTS, S/AS01 malaria vaccine in African children. N Engl J Med.

[31] The RTS SCTP (2012). A phase 3 trial of RTS, S/AS01 malaria vaccine in African infants. N Engl J Med.

[32] RTS, S Clinical Trials Partnership (2014). Efficacy and safety of the RTS, S/AS01 malaria vaccine during 18 months after vaccination: a phase 3 randomized, controlled trial in children and young infants at 11 African sites. PLoS Med.

[33] Garfinkel M (2014). A fresh look at self-plagiarism. Proc Am Assoc Adv Sci.

